# Recent Progress on Rare-Earth-Doped Upconversion Nanomaterials for Bioassay Applications

**DOI:** 10.3390/bios15060335

**Published:** 2025-05-23

**Authors:** Jiling Xu, Hengyuan Cao, Chenwei Wu, Ting Wang, Liheng Sun, Biao Dong

**Affiliations:** State Key Laboratory on Integrated Optoelectronics, College of Electronic Science and Engineering, Jilin University, Changchun 130012, China; jlxu23@mails.jlu.edu.cn (J.X.); caohy24@mails.jlu.edu.cn (H.C.); cwwu1921@mails.jlu.edu.cn (C.W.); wangting1921@mails.jlu.edu.cn (T.W.)

**Keywords:** rare earth, upconversion, biosensing, FRET

## Abstract

Rare-earth-doped upconversion nanoparticles (UCNPs) have been widely used in biological detection due to their unique anti-Stokes shift, stable chemical properties, tunable emission wavelengths, and low biotoxicity. However, their low fluorescence quantum yield remains a challenge. Constructing a high-performance detection platform based on UCNPs is therefore a critical consideration. Focusing on the biological detection applications of UCNPs, this paper introduces the fundamental principles of upconversion and the design of upconversion fluorescence probes. It then summarizes common strategies for enhancing upconversion luminescence and three biosensing platform formats: solution-based, strip-based, and plate-based. Finally, future directions for UCNPs in biological detection are discussed.

## 1. Introduction

Upconversion luminescence is an anti-Stokes process where sequential absorption of two or more low-energy photons leads to the emission of one high-energy photon. Bloembergen first observed this phenomenon in 1959 by exciting ZnS with 960 nm infrared light to produce green fluorescence [1]. Unlike quantum dots and organic fluorescent dyes, upconversion typically involves near-infrared excitation and visible light emission [2]. This unique optical property minimizes interference from biological autofluorescence, enabling high signal-to-noise ratios and deep tissue penetration. Due to these advantages, upconversion nanoparticles (UCNPs) have found broad applications in biological detection [3,4,5,6], bioimaging [7,8,9], and therapeutic applications [10,11,12,13].

**Figure 1:**
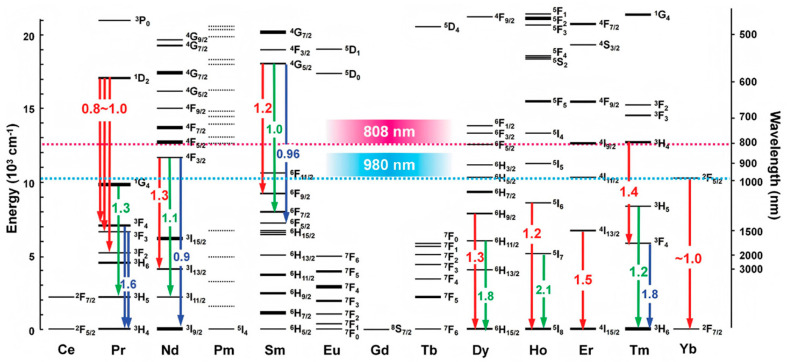
Abundant energy level transitions of positive Ln^3+^. Reproduced from Ref. [23], with permission from John Wiley and Sons; copyright 2019.

Rare-earth-doped upconversion materials represent a major category of luminescent materials. Rare earth elements comprise lanthanides, scandium, and yttrium. Lanthanide ions possess unfilled 4f energy levels. When doped into crystal matrices, the absence of a centrosymmetric crystal field causes 4f configurations to mix with opposite-parity wave functions, enabling 4f–4f intrashell transitions that result in light emission [14], as shown in Figure 1. These transitions endow Ln^3^^+^ ions with abundant energy levels. The shielding effect of the outer 5s5p electron shells makes lanthanide ions chemically stable and minimally affected by external environments [15]. The upconversion materials primarily consist of three components: host matrix, activator, and sensitizer. The matrix serves as a doping host for lanthanide ions without participating in light emission. An optimal matrix must meet three key requirements: (1) low lattice phonon energy to minimize non-radiative transitions, (2) appropriate lattice spacing matching dopant ions, and (3) excellent chemical stability [16,17]. Fluoride matrices, with their characteristically low phonon energy (~350 cm⁻^1^) and compatible ionic radii (Na^+^, Ca^2^^+^, Y^3^^+^), are particularly suitable for lanthanide doping [18]. Among various fluorides, NaYF_4_ has demonstrated superior performance as a host matrix for blue and green upconversion emissions [19]. The activator, serving as the luminescence center, undergoes energy level transitions to emit fluorescence. Different lanthanide ions exhibit distinct 4f energy level distributions, enabling multicolor emissions. For instance, Tm^3^^+^ and Er^3^^+^ are commonly used activators for blue and green light emission, respectively. The upconversion quantum yield initially improves with increasing activator concentration. However, excessive doping reduces interionic distances in the lattice, enhancing non-radiative transitions through the concentration quenching effect. Optimal doping concentrations are typically below 0.5% for Tm^3^^+^ and 3% for Er^3^^+^ [20]. Due to their weak near-infrared absorption, activators often require sensitizers. Sensitizers possess large absorption cross-sections for efficient energy transfer. Yb^3^^+^ (^2^F_7_/_2_→^2^F_5_/_2_ transition) absorbs 980 nm light, while Nd^3^^+^ (^4^I_9_/_2_→^2^H_9_/_2_ transition) preferentially absorbs 808 nm laser, thereby mitigating the water-heating effects associated with 980 nm excitation. Unlike activators, sensitizers require higher doping concentrations, typically around 20 mol% [21]. Rare-earth-doped upconversion materials exhibit several advantages, including excellent chemical stability, tunable emission wavelengths, narrow emission peaks, and low biotoxicity. However, their practical applications are constrained by relatively low fluorescence quantum yields, primarily due to the forbidden nature of 4f–4f transitions, inefficient energy transfer processes, and various non-radiative decay pathways [22]. Consequently, enhancing the luminescence intensity remains a key research focus for rare-earth-doped upconversion materials.

While recent reviews have comprehensively examined the luminescence mechanisms, synthesis approaches, and surface modifications of rare-earth-doped upconversion materials, some focusing on biosensing applications have been categorized by detection targets [24,25]. However, a systematic review addressing practical material applications remains lacking. This review examines recent advances and key publications regarding rare-earth-doped upconversion materials for bio-detection applications. We introduce the luminescence principle and material composition of upconversion, discuss the design principle and fluorescence enhancement strategy of fluorescence probes, and then trace the development of materials from the solution phase to the solid phase. Our analysis may provide valuable guidance for the design and application of upconversion fluorescent probes.

## 2. Principles of Fluorescence Probe Construction

The construction of fluorescent probes requires the effective conjugation of upconversion nanoparticles (UCNPs) to target molecules. As most bioassays are conducted in aqueous environments, the initial challenge involves modifying the hydrophobic surface of oleic acid-coated UCNPs to improve water solubility. Several surface modification strategies can be employed: (1) ligand exchange with amphiphilic molecules (e.g., cPEG, PAA, or PEI); (2) oxidation of oleic acid’s C=C bonds to carboxyl groups; or (3) encapsulation within an amino-functionalized SiO₂ shell. These modifications enhance water solubility, tune the surface charge, and introduce specific functional groups.

UCNP-targeted conjugation can be achieved through either electrostatic adsorption or covalent bonding. For small-molecule detection in vitro, electrostatic adsorption offers a straightforward approach. By tailoring the surface charge (e.g., amino groups for a positive charge or carboxyl groups for a negative charge), UCNPs with opposite polarity to the target can be selected for probe formation. However, this method lacks specificity and is susceptible to interference from environmental ions and pH variations, limiting its application to rapid in vitro assays. For biological macromolecules like proteins and nucleic acids, covalent coupling provides superior stability and specificity. Proteins contain accessible amino and carboxyl groups that can form amide bonds with complementary functional groups on UCNPs. A classical preparation method involves activating carboxyl-modified UCNPs with EDC/NHS reagents before mixing with target proteins in a buffer solution. Alternatively, amino-modified UCNPs can be crosslinked to proteins via glutaraldehyde. Similarly, nucleic acid conjugation requires terminal amino or carboxyl group modification for covalent attachment. These covalent approaches maintain binding efficiency under mild reaction conditions while ensuring probe specificity, particularly when combined with antigen–antibody recognition systems.

**Figure 2:**
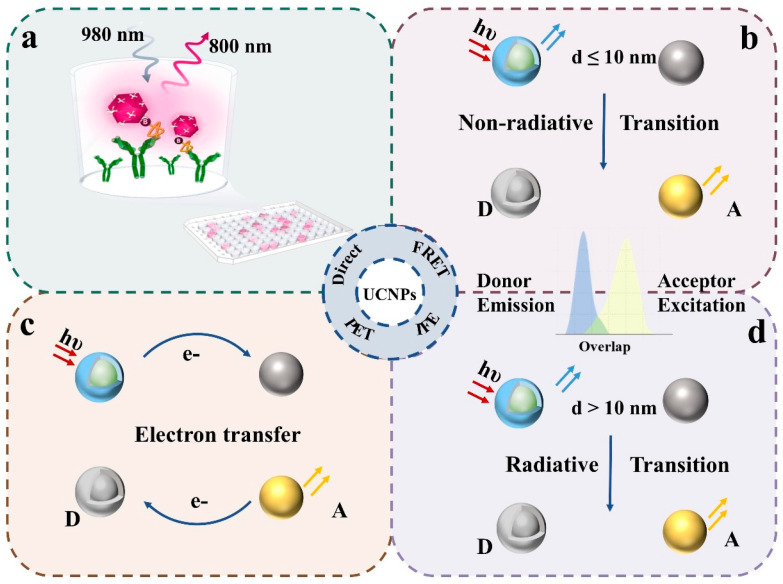
Construction of fluorescent probes: (**a**) direct detection; (**b**) Förster resonance energy transfer; (**c**) internal filter effect; (**d**) photo-induced electron transfer.

Although upconversion nanoparticles (UCNPs) can be conjugated to biomolecules in close proximity, their optical properties minimize interference with biomolecular absorption. Specifically, UCNP luminescence primarily occurs in the visible region, whereas most biomolecules absorb in the UV range. Consequently, UCNPs typically cannot directly modulate their fluorescence signals. Establishing a quantitative relationship between analyte concentration and system fluorescence intensity is therefore crucial. As shown in Figure 2, fluorescence probe design principles can be categorized into four types based on their detection mechanisms and signal generation approaches.

### 2.1. Direct Detection Method

Direct detection utilizes the luminescent properties of upconversion materials by conjugating fluorescent labels with target analytes, followed by fluorescence measurement after separation and enrichment for target localization or quantification. In contrast, this method has limited utility in complete solution-phase assays, primarily employed in imaging applications requiring fixed substrates. Al-Salihi et al. [26] synthesized NaYbF_4_@NaYF_4_:Er,Tm@NaYF_4_ NPs, and successfully realized fluorescent lifetime imaging of mouse cerebral vasculature using a triple-gate data acquisition method. Gorris’ team [27] used UCNP fluorescent probes to bind PSA antigens fixed on the pore plate by the sandwich method, and developed a new mode of simulating digital readings under fluorescence microscopy, raising the detection limit to sub-fM level. In addition to the integrated reading of combined detection, magnetic separation enhances sensitivity by combining target capture with enrichment. For instance, Song [28] employed Fe_3_O_4_ magnetic nanoparticles to bind clenbuterol hydrochloride, followed by aptamer-conjugated UCNPs to form MNP-CLB-UCNP complexes. Magnetic separation enabled detection at 0.304 ng mL^−1^.

### 2.2. Förster Resonance Energy Transfer (FRET)

Förster resonance energy transfer (FRET) is the most widely used fluorescence detection method. For effective FRET, three conditions must be satisfied: (1) the donor must be a fluorophore with a sufficiently long lifetime; (2) the donor’s emission spectrum must overlap with the acceptor’s absorption spectrum; (3) the donor–acceptor distance must be less than 10 nm. FRET detection primarily involves two approaches: modulating the donor–acceptor distance or selecting different acceptor types. The sub-10 nm dimensions of typical biomolecules (antigens, antibodies, and nucleic acids) make FRET particularly suitable for biological immunoassays. Signal generation occurs through two mechanisms: Aptamer-mediated acceptor binding that quenches upconversion fluorescence; and competitive target binding that restores donor fluorescence. In essence, the target concentration correlates with the number of donor fluorophores undergoing binding or dissociation events.

A representative example is the work by Lao et al. [29], who developed NaGdF_4_:Yb/Tm@NaYF_4_:Yb/Er nanoparticles with Tm^3^^+^/Er^3^^+^ co-doping for SARS-CoV-2 RNA detection. In this system, gold nanoparticles (Au NPs) served as acceptors to quench the Er^3^^+^ shell luminescence via FRET. Notably, the Tm^3^^+^-induced localized surface plasmon resonance (LSPR) of Au NPs was found to enhance the overall FRET efficiency. Although colloidal gold remains the most prevalent FRET acceptor, owing to its excellent chemical stability and broad absorption spectrum, its limited quenching efficiency has prompted the exploration of alternative acceptors. Recent studies have investigated various nanomaterials with larger surface areas or additional functionalities, including gold nanoflowers [30], MoS₂ nanosheets [31], MnO_2_ [32], and organic dyes [33]. These emerging materials represent promising directions for FRET-based detection systems. Therefore, optimal FRET system design requires careful consideration of both spectral overlap characteristics and specific signal requirements when selecting the appropriate acceptors. The choice of acceptor material should be guided by the intended application and desired detection performance.

### 2.3. Inner Filter Effect (IFE)

Unlike FRET, the inner filter effect (IFE) describes the reduction in fluorescence intensity when another substance in the system absorbs the donor’s emitted light. This phenomenon does not require close proximity between the donor and acceptor—spectral overlap alone is sufficient to induce IFE, significantly enhancing the system’s design flexibility and simplicity. IFE-based detection typically employs ratiometric fluorescence measurements, considering two aspects: On the one hand, whether there is a compound before and after that combines with the object to be measured can produce or eliminate absorption peaks that coincide with the emitted light. NaYF_4_:Yb,Er have become common UCNPs due to their emissions at 540 nm and 655 nm, which Zhang used to detect serum bilirubin [34], the original Fe^3+^ and sulfosalicylic acid (SSA) in the system. The combination of the two can quench green light at 540 nm, and the bilirubin and SSA compete to bind Fe^3+^ to restore green light. The I_G_/I_R_ readout value is used to quantify the green light. Similarly, mexiletine in serum can restore green light by electrostatic association with the dye Bengal Rose (RB) [35]. On the other hand, it can be considered whether the chemical reaction of the object to be tested can produce an absorbent. For example, glucose oxidase can degrade glucose to produce H_2_O_2_, thus oxidizing the gallic acid-Fe^2+^ complex (GA-Fe (Ⅱ)) to GA-Fe (Ⅲ), thereby quenching fluorescence [36]. More generally, the target analyte can also be detected indirectly by immobilizing it with other IFE-reactive components. For example, Wu et al. [37] developed an assay using aldehyde-functionalized magnetic nanoparticles to capture sulfadimethoxine (SDM), followed by binding with biotin-labeled aptamers and HRP. The SDM concentration was then determined through oxidized TMB quantification. Beyond using intrinsic upconversion emission ratios, ratiometric fluorescence can also be constructed by incorporating carbon dots as complementary emitters [38]. While IFE significantly broadens detection capabilities, its specificity remains inferior to immunoassay methods.

### 2.4. Photo-Induced Electron Transfer (PET)

Photo-induced electron transfer (PET) probes consist of a fluorophore and a quenching group. Upon light excitation, electron transfer occurs from the donor’s highest occupied molecular orbital (HOMO) to the acceptor’s lowest unoccupied molecular orbital (LUMO), resulting in fluorescence quenching [39]. This quenching mechanism demonstrates higher efficiency compared to FRET and IFE. Li et al. [40] identified o-quinone as an effective small-molecule quencher for upconversion luminescence (UCL) via PET, enabling alkaline phosphatase (ALP) detection. The system utilizes ALP-mediated hydrolysis of O-phospho-L-tyrosine to L-tyrosine, followed by enzymatic oxidation to generate the quencher L-dopa-o-quinone. Ye [41] developed crown ether-functionalized anthracene derivatives as quenchers for triplet–triplet annihilation upconversion systems. These probes achieved Mg^2^^+^ detection with a 2.52 μM limit through PET inhibition via Mg^2^^+^-crown ether complexation.

## 3. Luminescence Enhancement Strategy

Despite their versatile applications, UCNPs face an inherent challenge of low luminescence efficiency. The main reasons include a low transition probability of Ln^3+^ 4f electrons, poor light absorption and energy transfer efficiency, and a significant non-radiative transition. Current strategies for enhancing upconversion luminescence can be divided into two categories: internal regulation and external regulation. The internal control is based on the composition of the material to change the lattice symmetry of the matrix, the concentration of doped ions, the structure of the material, etc. External regulation is to connect or modify new substances on the surface of the material to play the role of light field regulation. These approaches address the fundamental limitations while providing pathways for performance improvement.

### 3.1. Structural Composition Regulation

**Figure 3:**
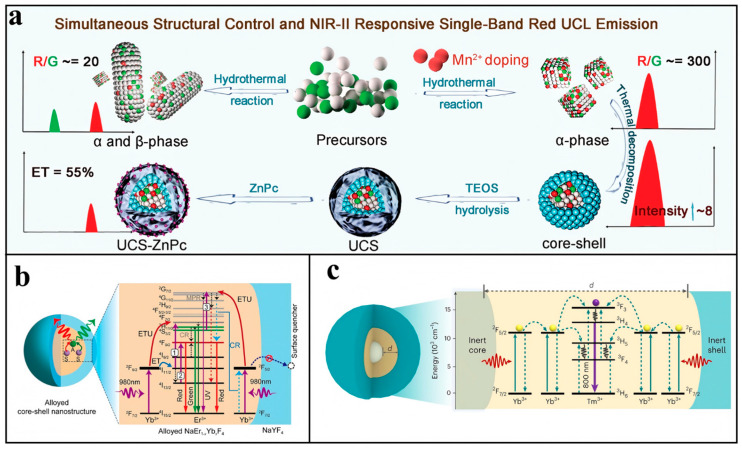
Structural control for upconversion enhancement: (**a**) Scheme of Er^3+^/Mn^2+^ co-doping to prepare UCNPs with NIR-Ⅱ-responsive single-band red UCL emission. Reproduced from Ref. [47], with permission from John Wiley and Sons; copyright 2022. (**b**) Schematic of energy transfer upconversion in the erbium–ytterbium alloyed core–shell nanoparticles under 980 nm excitation. Reproduced from Ref. [53], with permission from the American Chemical Society; copyright 2024. (**c**) Schematic illustration of simplified lanthanide energy levels as well as the involved upconversion processes in the core–shell–shell nanostructure. Reproduced from Ref. [58], with permission from Springer Nature; copyright 2024.

As previously discussed, the mixing of wave functions with opposite parity enables 4f–4f transitions in Ln^3^^+^ ions, while enhanced crystal field asymmetry significantly increases electric dipole transition probabilities. Doping with foreign ions to reduce local symmetry around lanthanides thus represents an effective approach for luminescence enhancement [42]. For oxide and fluoride substrates, Mn^2+^, Fe^3+^, Cu^2+^, Ni^2+^, Mg^2+^, Cr^3+^, and other transition-metal ions have been reported to be doped successfully to improve the luminescence intensity [43,44,45,46]. Figure 3a [47] shows a NIR-Ⅱ-responsive red single-band material by co-doping NaLuF_4_ with Er/Mn. The experiment found that when the content of Mn^2+^ increased from 0 to 40%, the upconversion intensity increased by 10 times, and the I_R_/I_G_ increased significantly from 20 to about 300. The same phenomenon was also found in doping Mn^2+^ in Ba_2_Sc_0.67_Yb_0.3_Er_0.03_AlO_5_ phosphor [48], which proved that the inclusion of Mn^2+^ can enhance the luminescence of Er^3+^ through the energy transfer between Mn^2+^ and Er^3+^ and the energy transfer between Er^3+^ and the defect band.

The luminescence efficiency of upconversion materials initially improves with increasing lanthanide ion concentration (activators and sensitizers). However, excessive doping reduces interionic distances, inducing cross-relaxation that ultimately decreases the material’s brightness [49]. In response to this concentration quenching phenomenon, it was found that increasing the laser power [50,51] and constructing the inert shell could have a strong brightness at a relatively high activator concentration. However, high-power laser often fails to meet the needs of practical applications (such as biological monitoring). Zhang Hong’s team found that at a low temperature (40 K), the upconversion emission intensity of NaErF_4_@NaYF_4_ increased sharply by 170 times, compared with NaYF_4_:20%Yb,2%Er@NaYF_4_. Low temperatures limit the cross-relaxation between Er^3+^, and high doping can be applied in low-temperature imaging and temperature sensing [52]. In addition, the Zhou Bo group [53] designed the alloying NaEr_0.01_Yb_0.99_F_4_@NaYF_4_, which effectively reduces the distance between Er^3+^ and Yb^3+^ and promotes energy transfer, as shown in Figure 3b. Compared with the traditional doping content, under the laser power of 0.26 W/cm^2^, the emissions of green light and red light are increased by 2786 times and 89 times, respectively. This provides a new idea for high blending.

UCNPs exhibit significant surface quenching effects due to their high surface-to-volume ratio, where excitation energy from internal ions migrates to surface defects or is quenched by ligands and solvents. The external coating of the shell is a common surface passivation method. The most common shell types are homogeneous inert rare-earth shells [54,55] and SiO_2_ [56,57], among which SiO_2_ has good water dispersion and can also play the role of surface modification. The choice of shell material and structure, and the distribution of doped ions in the core–shell structure, will affect the brightness of the material. As shown in Figure 3c, Chen’s team constructed a sandwich nanocrystalline structure, β-NaYF_4_@NaYF_4_:Yb/Tm@NaYF_4_ [58]. Using the effective confined isolation protection of the inert material in the core and outer shell, they demonstrated the size-dependent effect of the quantum efficiency of the material’s upconversion by precisely regulating the thickness of the intermediate layer (1.2–13 nm). The epitaxial growth of ScF_3_ matched with the NaYF_4_ (111) crystal face on the α-NaYF_4_:Yb/Ln surface will give the material positive/negative thermal expansion properties, and unlike the traditional thermal quenching conversion, UCL will be enhanced with the increase of temperature [59].

### 3.2. Dye Sensitization

Rare-earth-doped upconversion materials exhibit limited light absorption cross-sections. In comparison, organic dyes demonstrate superior light-harvesting capabilities, with absorption cross-sections 10^3^–10^4^ times greater than lanthanide ions [60,61]. To address this limitation, researchers developed dye-sensitized upconversion systems by conjugating organic dyes to UCNP surfaces as light-harvesting antennas.

**Figure 4:**
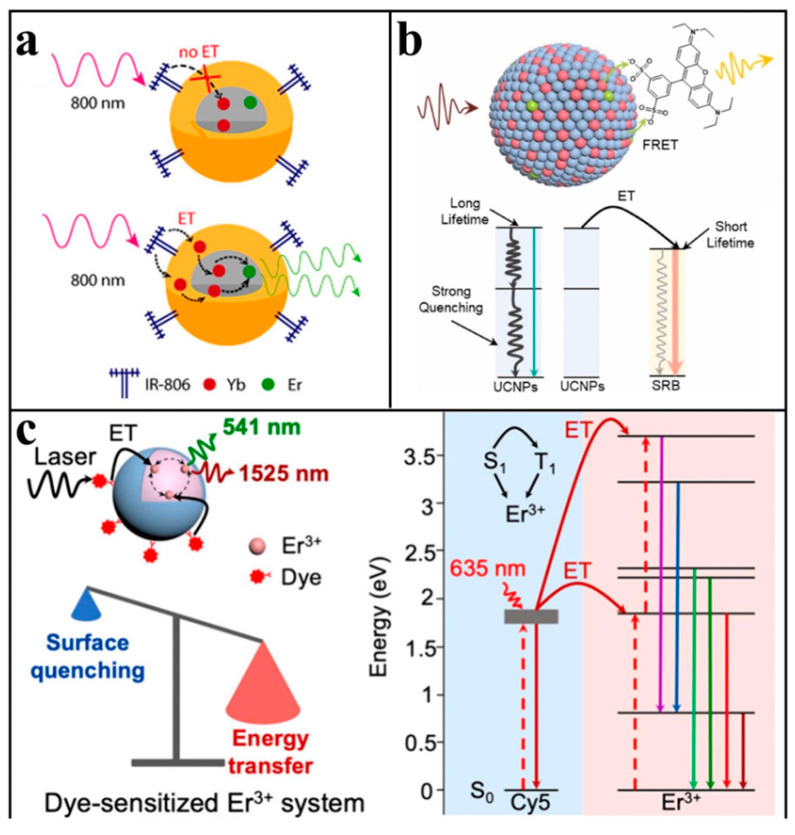
Dye sensitization for upconversion enhancement: (**a**) Schematic diagram of the dye-enhanced upconversion luminescence principle. Reproduced from Ref. [61], with permission from the American Chemical Society; copyright 2016. (**b**) Schematic representation of organic dyes as receptors for sensitized upconversion emission. Reproduced from Ref. [63], with permission from Elsevier; copyright 2025. (**c**) Schematic illustration of energy transfer for direct sensitization of Er^3+^ by Cy5. Reproduced from Ref. [64], with permission from the American Chemical Society; copyright 2024.

As shown in Figure 4a, the usual pathway for dye delivery is first to the sensitizer and then to the activator. The effect of dye sensitization depends on the degree of spectral overlap between the dye and Ln^3+^ and the distance between the dye and the receptor, so there are limitations in dye selection. These parameters impose strict selection criteria for optimal dye performance.

Meng et al. [62] modified NaYF_4_:20%Yb,1%Ho@NaLuF_4_:10%Yb,40%Nd nanoparticles with excellent luminescence properties under 808 nm excitation by ICG. ICG transferred the energy of the 808 nm light source to Nd^3+^, and Nd^3+^ further transferred the energy to Yb^3+^ and finally to the luminescence center Ho^3+^. The experiment found that the sensitization of ICG enhanced the luminescence of the material at 540 nm by about 80 times. At the same time, dyes can also be used as receptors to enhance the upconversion efficiency. For example, Rhodamine sulfonyl B (SRB) is connected to the surface of NaGdF_4_@NaYF_4_: Gd^3+^, Yb^3+^,Er^3+^, and SRB will absorb the emitted light at 540 nm of the material into its own light at 645 nm. Researchers found that dyes acting directly as FRET receptors can reduce the non-radiative relaxation of the system, as seen in Figure 4b. The surface modification of SRB results in an energy transfer efficiency of up to 98.8%, and the luminescence intensity of SRB as a receptor is 4.6 times that of the UCNPs at a 541 nm emission reduction [63]. Liu’s team [64] proposed a new strategy for dye direct sensitization of the Er^3+^ emission. Different from traditional dye sensitization receptors Yb^3+^ and Nd^3+^, Er^3+^ can absorb from ultraviolet to near-infrared. The researchers used a series of cyanine dyes with absorption bands coincident with Er^3+^ as antenna molecules and found that they all play an enhanced role. Cy5, with the largest spectral overlap, enhanced UCL by a factor of 1942, which provided the idea for polychromatic excited dye sensitization; the corresponding process is reflected in Figure 4c.

### 3.3. Local Field Control

To enhance upconversion luminescence (UCL), two primary external modulation approaches exist alongside internal rare-earth material modifications: surface plasmon resonance (SPR) and photonic crystal modulation. These local field modulation strategies offer broader applicability and operational convenience compared to component-specific modifications. By increasing the photonic state density at the material surface, they effectively boost UCL efficiency.

**Figure 5:**
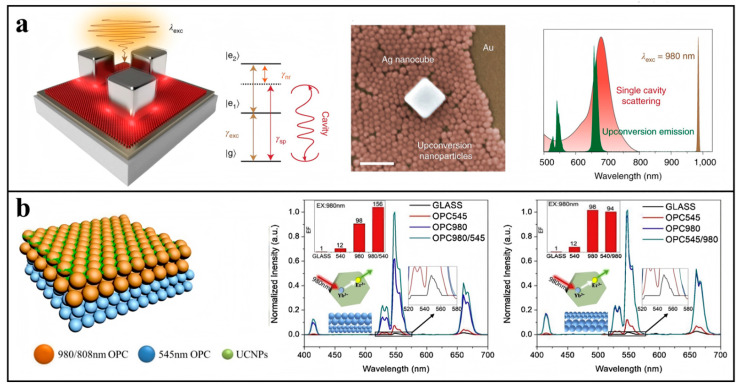
Local field control for upconversion enhancement: (**a**) Structural design of plasma cavity-coupled upconversion nanoparticles (UCNPs) and schematic SEM and photoluminescence spectra. Reproduced from Ref. [66], with permission from Springer Nature; copyright 2019. (**b**) Schematic structure of a double-stopband photonic crystal and the enhancement of a photonic crystal film with the excitation field emitting field in the opposite position. Reproduced from Ref. [69], with permission from Elsevier; copyright 2021.

Localized surface plasmon resonance (LSPR) in noble metals involves collective electron oscillations at the metal surface under light excitation, generating propagating surface plasmons that create enhanced electromagnetic fields. The Purcell effect predicts optimal luminescence enhancement when the emitter is positioned within an optical cavity and the cavity resonance matches the emitter’s emission frequency [65]. Many works have been conducted to enhance UCL with various forms of precious metals or cavities with different structures; e.g., Figure 5a shows an optical cavity with silver nanocubes arranged on a gold film and which fills the nanoparticle gaps with UCNPs designed by Liu Xiaogang’s team [66], obtaining a 166-fold enhancement factor of luminescence at a low excitation power. In order to realize the resonant enhancement of the excitation and emission light at the same time, Xu et al. [67] designed a UCNPs/MgF_2_/Ag optical structure and controlled the coupling of light by adjusting the two-dimensional grating period, which confirms that the UCL can be maximally enhanced when the grating is coupled with the excitation and emission wavelengths of UCNPs at the same time.

Photonic crystals are periodic dielectric structures with alternating refractive indices that exhibit photonic bandgap properties. When incident light wavelengths match the bandgap, total reflection occurs, producing characteristic structural colors. The bandgap position depends primarily on the structural unit size (typically microspheres), enabling tunability through material dimension control. The main measure for photonic crystal enhancement of fluorescent materials is to make the bandgap position coincide with the excitation wavelength or emission wavelength. The former allows the excitation light to be repeatedly reflected and scattered between the gaps of the photonic crystal, which enhances the energy density of the excitation light; the latter plays an enhancement effect by modulating the direction of emission of the emitted light. Ju [68] designed an opal with a bandgap of 450 nm for the poly(methyl methacrylate) (PMMA) deposited photonic crystals to enhance the blue UCL of Tm^3+^ for glucose detection. As depicted in Figure 5b, our group explored the enhancement of the luminescence of UCNPs by double-stopband-bilayer opal photonic crystals and found that the modulation of the excitation field of the photonic crystal plays a dominant role, and that the combined action of the excitation and emission fields can have a superimposed enhancement effect, and that double-forbidden-band photonic crystals enhanced luminescence at 540 nm by a factor of 156 for NaYF_4_: Yb,Er [69]. Similarly, double-forbidden-band anti-opal photonic crystals have been applied in anti-counterfeit markers with bright fluorescence [70].

## 4. Platform for UCNPs in Bioassays

### 4.1. Solution-Phased Detection

Solution-phase detection represents the most prevalent fluorescence assay format, offering homogeneous reaction conditions and stable detection sensitivity. However, this approach faces challenges, including operational complexity and a lack of portability.

The development direction of the upconversion solution form of detection is most notably to enhance the luminescence intensity of UCNPs, such as the structural modulation of the material itself and plasma coupling, mentioned above, which will not be reintroduced in this section. It is also possible to consider increasing the binding sites of the reaction and the probability of the reaction occurring, etc. The following highlights several innovative approaches in this field.

Hou et al. [71] first introduced the atom transfer radical polymerization (ATRP) reaction, which refers to the phenomenon of reversible transfer of an initiator between active and dormant species in the presence of a transition-metal complex. The modification of antibodies and UCNPs with initiator and reactive monomers, respectively, could make UCNPs accumulate on the surface of magnetic beads in large quantities by ATPR, which could enhance the fluorescence intensity. The system achieved a 38.7 fg/mL detection sensitivity for CYFRA21-1. Two-dimensional materials with a large specific surface area, such as MXene, are favorable for increasing the reaction sites. For example, Hao immobilized red- and blue-emitting UCNPs on Nb_2_CT_x_ MXene [72], and the double-helix structure formed in the presence of the target was repelled away by the negatively charged MXene, and the FRET was invalidated so as to restore the fluorescence, which realized the simultaneous detection of the two substances and enhanced the accuracy of the detection. Their team also used Au-Au dimer as a novel FRET bursting agent [73]; a dimer form that generates a stronger electromagnetic field, improves the bursting efficiency, and extends the working distance. The accurate detection of long-chain oligonucleotides was realized, and the detection limit was even lower than the aM level, which provided a new idea for conventional FRET detection.

### 4.2. Lateral Flow Chromatography

Test strips represent a cost-effective biosensing platform with multiple advantages, including operational simplicity, rapid detection, user-friendliness, and field applicability, leading to their commercialization in immunoassays [74]. A standard test strip consists of a sample pad, conjugate pad, nitrocellulose membrane (with test [T] and control [C] lines), absorption pad, and backing card. In immunoassays, the T-line is coated with target-specific antibodies, while the C-line contains universal antibodies. After adding the sample, the target analytes will bind with detection antibodies in the conjugate pad, and the complexes migrate to the T-line via lateral flow. Unbound antibodies reach the C-line for quality control. Colloidal gold is the most common indicator for test strips and can be read directly with the naked eye. Still, this indication method has the problem of low sensitivity [75] and is typically applicable only for qualitative binary (positive/negative) analysis. Compared with conventional colorimetric methods, fluorescence reporter molecules can greatly improve detection sensitivity up to 100–10,000 times and better meet the quantitative requirements [76]. Upconversion nanoparticles (UCNPs) offer distinct advantages for bioassays, primarily due to their near-infrared (NIR) excitation that avoids the autofluorescence background associated with conventional UV excitation. Furthermore, multicolor UCNPs enable the simultaneous detection of multiple analytes. For high-precision applications, UCNP-based lateral flow assays present an optimal solution. While the inability to read UCNP signals with the naked eye increases operational complexity, this limitation can be overcome by employing a simple laser flashlight coupled with smartphone-based grayscale analysis, maintaining portability while digitizing the measurement results for point-of-care testing (POCT). Notably, several biotechnology companies, including Hotgen and Germany-based Miltenyi Biotec, have developed handheld UCNP immunoassay analyzers to facilitate the large-scale implementation of this technology. Recent advances demonstrate the successful integration of upconversion nanoparticles with test strips, achieving enhanced performance for biological detection.

**Figure 6:**
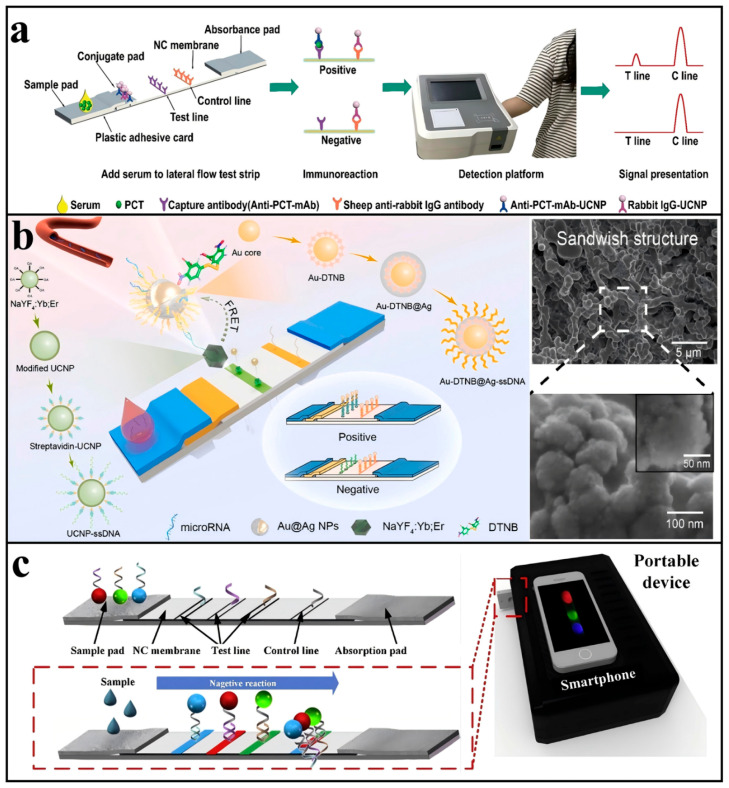
Biological detection on test strips: (**a**) Schematic illustration of NIR to NIR lateral flow detection based on upconverted nanoparticles. Reproduced from Ref. [79], with permission from the American Chemical Society; copyright 2020. (**b**) Schematic diagram of the fluorescence and Raman dual-mode sensor of the FRET principle on the test strip and SEM image of the sandwich structure formed on the test strip. Reproduced from Ref. [81], with permission from Elsevier; copyright 2024. (**c**) Schematic illustration of LFAA for simultaneous multiple target detection. Reproduced from Ref. [83], with permission from Elsevier; copyright 2018.

The detection effect of the test strip is mainly affected by four aspects: target acquisition, signal conduction, signal separation, and signal analysis [77]. Jin [78] comprehensively optimized the signal reading device, the release efficiency of the UCNPs probe, the change in fluorescence intensity caused by the size of UCNPs, and the reference value of line C to reduce the deviation. The superimposed effect of multiple links made the detection limit of H. pylori nucleic acid reach 25 fM, a 105-fold increase. In view of the signal separation process, as shown in Figure 6a, Ji [79] innovatively took the emission light of NaYF_4_:Yb^3+^,Tm^3+^@NaYF_4_ at 800 nm as the signal, so that the excitation wavelength (980 nm) and emission wavelength of the material are both in the first biological window, avoiding the interference of biological self-fluorescence and colored plasma scattering. The detection limit of procalcitonin in serum reached 0.03 ng/mL. For signal analysis, a portable in situ detection biosensor combined with a smartphone has been invented [80], which overcomes the traditional lengthy pre-processing and fuzzy qualitative results, in which the computer vision algorithm improves the sensitivity and robustness of quantitative results, and the accuracy of quantitative detection results is increased by 17.4% compared with the standard quantitative detection process. The detection limit of methamphetamine can reach 0.1 ng/mL within 20 s.

The strip combines the advantages of solid and liquid sensors. On the one hand, it is easy to carry and easy to operate. On the other hand, due to the supporting role of the three-dimensional fiber structure of the cellulose nitrate film, the strip can provide a contact environment close to solution, which solves the problem of an insufficient reaction area and limited contact in-plane detection. Chen et al. [81] took NaYF_4_:Yb^3+^ and Er^3+^ UCNPs as fluorescence reporter molecules, and pre-coated them on the T-line of the nitrate cellulose film by drops. Using the FRET mode, Au NPs were replaced by Au-DTNB@Ag with Raman signals. With an increase in target material, the number of Au@Ag particles accumulated on the T-line increased, the fluorescence signal weakened, and the Raman signal strengthened (Figure 6b). This method was used to detect microRNA-21—a marker of periodontitis in saliva—and reached the dynamic range of 2 nM–1 fM, which greatly improved the detection sensitivity in this field. This method provides a good strategy for the simple and sensitive application of test strips.

The capillary action of the strip allows the liquid to flow completely from one side to the opposite side, which means that it can be spatially partitioned, allowing different targets to be combined in different locations and supplemented by upconversion reporter molecules of different colors, enabling simultaneous detection of multiple substances. For example, Sahar et al. [82] first explored the relationship between the content and ratio of CD44 and FKBPL proteins in serum and preeclampsia. The authors used the same system to construct their own test strip for these two proteins. Although there was no cross-reactivity and the sensitivity reached the pg/mL level, the operation was relatively complicated. Jin et al. [83] synthesized red, green, and blue upconversion fluorescent probes, designed a structure of test paper containing three T-lines and one C-line, as illustrated in Figure 6c, and realized the simultaneous detection of three different types of analytes, Hg^2+^, ochratoxin A, and Salmonella, in water samples. However, the number of lines was limited by the limited diffusion distance of the samples on the strip; thus, Miikka [84] chose to place both the Er^3+^-doped green light upconversion probe and Tm^3+^-doped blue light upconversion probe on the T-line to simultaneously test CA125 and CA15-3 proteins. Guo et al. [85] designed and synthesized NaErF_4_:Yb^3+^,Tm^3+^@NaYF_4_:Yb^3+^@NaNdF_4_:Yb^3+^ UCNPs with orthogonal emission properties, which produced red fluorescence at a 980 nm excitation and green light at an 808 nm excitation, achieving orthogonal emission. The intensity ratio (IG/IR) served as an internal reference, eliminating the need for a control line (C-line) and thereby simplifying the strip design.

Given the versatility of test strips as platforms for diverse sensing modalities, we conducted a comprehensive comparison between UCNP-based detection systems and conventional reporter molecules for multiple bioanalytes. The comparative performance metrics are systematically summarized in Table 1. Upconversion nanoparticles (UCNPs) demonstrate broad applicability for detecting diverse biological targets. Compared with the colorimetric and electrochemical methods, UCNP-based detection offers superior sensitivity while not matching the ultra-high sensitivity of Raman spectroscopy; rather, it provides a significantly wider dynamic range and better adaptability.

### 4.3. Plate

Plate-based biosensors confine the sensing process to a planar surface, offering the portability advantages of solid-state devices while typically exhibiting lower detection sensitivity. Research efforts, therefore, focus on two key challenges: (1) enhancing localized fluorescence intensity and (2) developing more sensitive detection modalities within these constrained areas.

**Figure 7:**
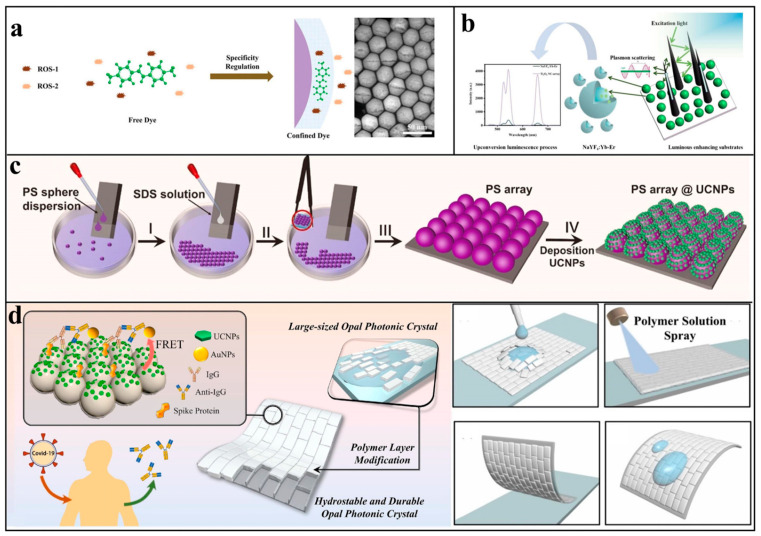
Biological detection on different types of planes: (**a**) Schematic illustration of self-assembled monolayer UCNPs plates for specific selective detection of ROS. Reproduced from Ref. [95], with permission from John Wiley and Sons; copyright 2023. (**b**) Schematic diagram of Ti_3_O_5_ nanocone array with structural constraint effect and LSPR-enhanced upconversion luminescence. Reproduced from Ref. [99], with permission from the American Chemical Society; copyright 2024. (**c**) Structural diagram of the UCNP plate deposited on the PS microsphere array. Reproduced from Ref. [100], with permission from Elsevier; copyright 2024. (**d**) Schematic diagram of the detection of COVID-19 by highly hydrostable opal photonic crystal film deposited with UCNPs. Reproduced from Ref. [101], with permission from Elsevier; copyright 2023.

Beatriz et al. [94] immobilized cysteine- and antibody-modified nanoparticles (UCNPs@Cys-Ab) on quartz substrates via layer-by-layer self-assembly using sodium dodecyl sulfate. Optimal signal intensity was achieved with six assembly layers, enabling Escherichia coli detection across a dynamic range of 2 × 10^3^–10^6^ CFU/mL, with a detection limit of 34 CFU/mL. Chen Guanying’s team [95] developed ROS-responsive IR820 dye-sensitized UCNPs (Figure 7a), where steric hindrance restricted IR820 absorption exclusively to ·OH. Under 808 nm irradiation, ·OH quenched the upconversion monolayer fluorescence while the 980 nm emission remained unaffected, allowing ratiometric detection of endogenous H₂O₂ with a 0.1 nM detection limit. In addition to self-assembly, UCNPs can also be deposited on other polymer substrates. PDMS is a common solid substrate. Ouyang et al., for example, anchored UCNPs on amino-functionalized PDMS substrate [96] to achieve a sensitive detection of malachite green of 0.029 ng/mL. Similarly, Rong [97] encapsulated UCNPs in PDMS to create a solid-state sensor suitable for testing actual samples of acrylamide produced during frying, with a detection limit of 1 nM. In addition, Gorris has performed a number of assays using a 96-well plate as a substrate using the upper conversion link immunosorbent assay (ULISA). Similar to ELISA, ULISA uses UCNPs instead of traditional enzymes and fluorescence signals instead of colorimetric signals that are generated by enzymatic reactions, achieving better sensitivity. Compared with the analog ULISA, which is based solely on luminous intensity, the digital ULISA, which counts light spots under a fluorescence microscope, can further improve the sensitivity by 10 times [98].

For the above example of UCNP itself or other polymers as solid substrates, the substrate itself does not have the enhancement effect; the following describes some signal enhancement substrates, mainly including the plasma effect and photonic crystal modulation. In view of the weak plasma effect of defective semiconductors, Xu et al. [99] designed the Ti_3_O_5_ nanopyramid array substrate (Figure 7b). By using the sharp structure of the nanopyramid, the excited light can be reflected several times inside the array and coupled with the surface plasma of the material itself, thus increasing the green and red luminescence of the upper UCNPs by 32 times and 40 times. It shows superior semiconductor enhancement performance and confirms that the special structure of the substrate can modulate the light field. Similarly, Wu [100] prepared the PS microsphere array @UCNPs’ flexible film by the self-assembly method (Figure 7c). Compared with the plane, the UCL strength of the array was increased by 10 times, and similar results were obtained by FDTD theoretical simulation, which was used to detect acetic acid gas, reaching a detection limit of 5.2 ppm. This enhancement was interpreted as Mie scattering on the surface of the PS microsphere. The photonic crystal is also an ordered arrangement of a microsphere array, which is different from a single layer. Furthermore, it behaves as a microsphere array in three-dimensional space. Intuitively, there is a photonic band gap in the transmission spectrum so that the enhancement factor can be further increased to tens of times. Our group successfully prepared a flexible film of photonic crystals by spraying polymers. As shown in Figure 7d, this method uses polymers to fill the fragmentary gaps in photonic crystals, acting as a bond, which solves the problem that photonic crystals are easily broken and do not have water stability on a macro level; thus, it can be used to detect biological samples. The detection sensitivity of 0.1 ng ·mL^−1^ for COVID-19 was achieved [101]. Photonic crystal enhancement is also used to detect glucose [68]. The author cleverly utilized the characteristic that H_2_O_2_, the oxidation product of glucose, can react with the quenching agent MnO_2_ to generate Mn^2+^, and constructed a fluorescence colorimetric dual-mode detection platform. The combination of photonic crystals and the metal plasma effect is also a common idea. Our research group has long explored the enhancement effect of gold bars deposited on opal photonic crystals [102], and found that when the SPR matches the excitation light spectrum, the plasma enhancement is the most significant, which can increase UCL by 3 orders of magnitude. Similarly, Chu [103] developed a flexible upconversion optical transducer using a dendritic gold inverse opal framework. This design demonstrated 48-fold luminescence enhancement at ultralow laser power, confirming synergistic interactions between photonic bandgap effects and localized surface plasmon resonance (LSPR). This device shows promising potential for implantable optogenetic applications.

### 4.4. Linkage with Other Technologies

To enhance the sensitivity and accuracy of bio-detection, in addition to enhancing the luminescence performance of UCNPs themselves, we can consider increasing the target analyte quantities and constructing multimodal detection modes. Some functional materials, such as chalcogenide quantum dots, nano-enzymes, and magnetic materials, have been developed into UCNP-based nanocomposites. In addition, nucleic acid amplification techniques such as catalytic hairpin self-assembly are also commonly used as a means of design. Table 2 summarizes the representative applications of these approaches.

## 5. Summary and Prospect

This paper introduces the composition of rare-earth-dope upconversion nanoparticles (UCNPs) based on upconversion luminescence principles. Focusing on bio-detection applications, we summarized four fluorescence probe design strategies as follows: direct detection, Förster resonance energy transfer (FRET), inner filter effect (IFE), and photo-induced electron transfer (PET). To overcome UCNPs’ low luminescence efficiency, three luminescence enhancement means, namely, structure composition modulation, dye sensitization, and local field modulation, are summarized. The structural composition modulation includes the doping of other metal ions, the proportion modulation between different compositions, and the construction of the core–shell structure; the local field modulation can be subdivided into the plasma resonance effect of the noble metals or semiconductors and the modulation effect of the photonic crystals on the excitation or emission light field. We also reviewed the bioassay work from recent years in the form of practical applications of UCNP detection, which is divided into three categories: solution environment, test strips, and plates. This review provides new perspectives for UCNP biosensing applications, highlighting both fundamental principles and practical implementations.

Despite their advantage of low biofluorescence background, UCNPs face several challenges, such as the photothermal effect of 980 nm excitation may cause tissue damage, while limited binding sites restrict detection capabilities. Recent research reveals three key development trends as follows: (1) The preparation of small and bright UCNPs: UCNPs with small and homogeneous particle sizes can provide more binding sites and easier access to biological tissues, but small particles increase the percentage of bursts and are often insufficiently bright; the modification of ligands and the excitation light source is a feasible strategy in this regard. (2) Construction of a multimodal detection system: The association of UCNPs with other materials, such as chalcogenides and nano-enzymes, can make the system’s characteristics more diverse. Relative to fluorescence unimodal detection, the introduction of detection modes, such as electrochemistry and Raman, can make the detection results more accurate, expanding the scope of application. (3) Development of corresponding detection equipment: How to transfer from laboratory research to a practical application is an important development direction for UCNPs in the future, and the development of a small portable device integrating a laser and a reading device is necessary.

## Figures and Tables

**Table 1:** Performance comparison of UCNPs with other biosensors (paper-based).

Biomass Category		Sensing Method	LOD	Linear Range	Ref
Organic small molecules	Methamphetamine	UCNP-based	47.25 pg/mL	5.012 × 10–5.012 × 10^5^ pg/mL	[86]
	Acetamiprid	nanoenzymatic colorimetry	25 cells/mL	10^2^–10^4^ cells/mL	[87]
Antigen	PSA	UCNP-based	0.65 ng/mL	1.5–80 ng/mL	[88]
	PSA	SERS	0.08 ng/mL	0–400 ng/mL	[89]
Nucleic acid	miRNA-21	UCNP-based	1 fM	1 fM–2 nM	[81]
	H1N1	nanoenzymatic colorimetry	0.02 nM	0.02–50 nM	[90]
Protein	FKBPL	UCNP-based	10 pg/mL	0.025–100 ng/mL	[82]
	Insulin	electrochemical	690 pM	0–5 nM	[91]
Microorganism	Avian influenza virus	UCNP-based	10^3.5^ EID_50_/mL	10^3.5^–10^5.5^ EID_50_/mL	[92]
	Salmonella typhimurium	QDs	25 cells/mL	10^2^–10^4^ cells/mL	[93]

**Table 2:** Application of biosensors based on upconversion composite materials.

Type	Materials	Target	LOD	Mechanism	Ref.
Perovskite	Donor: CsPbBr_2_I@UCNP Acceptor: NiMn-LDH/CdS	Malathion	4.8 fg/L	FRET PET	[104]
	Donor: UCNP@SiO_2_ Acceptor: CsPbX_3_	miRNA-155	73.5 pM	FRET	[105]
Nano-enzyme	Donor: UCNP@SiO_2_/CeO_2_ Acceptor: MGO	S-cDNA	UCL: 320 fM Colorimetric: 28.4 pM	FRET	[106]
	Donor: UCNPs Acceptor: reaction product	Hypoxanthine	0.69 mg/L	IFE	[107]
	Donor: UCNPs Acceptor: oxTMB	Ofloxacin	UCL: 0.048 μg/kg Colorimetric: 0.165 μg/kg	IFE	[108]
	Donor: UCNPs Acceptor: Fe_3_O_4_@NPC	Aflatoxin B1	0.56 pg/mL	FRET	[109]
Magnetic material	Donor: UCNPs Acceptor: Fe_3_O_4_@Au	SARS-CoV-2	2.1 pg/mL	FRET	[110]
MOF	Donor: UCNP@UiO-66-NH_2_ Acceptor: NiSx	H_2_O_2_	0.15 μM	IFE	[111]
	Donor: 30%Yb^3+^-EuMOF Acceptor: MG	Malachite green	36.33 nM	FRET	[112]
Chiral material	Donor: UCNPs Acceptor: Cu_x_OS	H_2_S	UCL: 43 μM CD: 22 μM	FRET	[113]
CHA	UCNPs-ssDNA-BHQ1	PSA	0.648 pg/mL	FRET	[114]
	UCNPs@DNAzyme-BHQ1	miRNA-21	31 fM	FRET	[115]

## Data Availability

Not applicable.
